# Characteristics and recurrence of pterygium in Saudi Arabia: a single center study with a long follow‐up

**DOI:** 10.1186/s12886-021-01960-0

**Published:** 2021-05-11

**Authors:** Waleed Alsarhani, Saeed Alshahrani, Mahmood Showail, Nawaf Alhabdan, Osama Alsumari, Abdullah Almalki, Abdulaziz Alsarhani, Adel Alluhaidan, Bader Alqahtani

**Affiliations:** 1 Department of Ophthalmology and Vision Sciences, University of Toronto, Toronto, Ontario Canada; 2 Department of Ophthalmology, King Faisal Specialist Hospital and Research Centre, Riyadh, Saudi Arabia; 3 College of Medicine, King Saud bin Abdulaziz University for Health Sciences, Riyadh, Saudi Arabia; 4 Department of Ophthalmology, Faculty of Medicine, King Abdulaziz University, Jeddah, Saudi Arabia; 5 College of Medicine, King Saud University, Riyadh, Saudi Arabia; 6 Department of Ophthalmology, Ministry of the National Guard – Health Affairs, Riyadh, Saudi Arabia; 7 King Abdullah International Medical Research Center, Riyadh, Saudi Arabia; 8 Cornea, Anterior Segment and Refractive Surgery Department, University of Ottawa Eye Institute, Ottawa, Ontario, Canada

**Keywords:** Pterygium, Conjunctiva, Recurrence

## Abstract

**Background:**

This study described the clinical features of patients with pterygium and analyzed the recurrence rate of conjunctival autografting alone, conjunctival autografting combined with intraoperative mitomycin C, and amniotic membrane grafting.

**Methods:**

A retrospective cohort study of primary pterygium was conducted between January 2017 and February 2020. Factors associated with pterygium severity and recurrence were analyzed by univariate analysis and logistic regression models.

**Results:**

The study included 292 patients with an average age of 53.3 ± 14.1 years, while the number of operated cases was 94. Pterygia involving the cornea were observed in 55 % of the cases. The overall rate of recurrence for the three procedures was 17 %. The average time of recurrence was 14.2 ± 11.9 months, with 37 % of the recurrences occurring after the first year. The only factor associated with a significant risk of recurrence was dry eye disease in both univariate (*p* = 0.021) and multivariate analysis (*p* = 0.026). The recurrence rates following conjunctival autografting with and without mitomycin C were 15.6 and 15.8 %, respectively. The recurrence rate following the amniotic membrane graft was  twofold (OR= 2.02)  (27 %) that following the conjunctival autograft (15.8 %).

**Conclusions:**

The only factor associated with the recurrence of pterygium was dry eye disease. More than one-third of recurrences developed after the first year, which stresses the importance of a long follow-up. The recurrence rate in our study following conjunctival graft was slightly higher compared to the literature mainly due to differences in study areas, populations, and follow-up periods.

## Background

A pterygium is a wing-shaped fibrovascular tissue extending from the bulbar conjunctiva into the cornea [1]. While pterygium can be asymptomatic, patients may complain of tearing, foreign body sensation or redness. When a pterygium enlarges and encroaches on the cornea, visual disturbance may result from astigmatism, direct visual axis obscuration, or even diplopia caused by limited extraocular motility [2]. The etiology of pterygium is multifactorial; nonetheless, increased ultraviolet (UV) light exposure is the most likely pathogenetic factor for disease development. UV light exposure results in oxidative stress and the release of cytokines and growth factors, eventually inducing cellular proliferation [3, 4]. This also explains why lower geographic latitude, outdoor activity, and living in rural areas and countries close to the equator are associated with an increased prevalence of pterygium [5, 6]. The worldwide prevalence of pterygium was reported in two meta-analyses performed in 2013 and 2018 and was found to be 10.2 and 12 %, respectively [5, 7]. Despite the fact that dry weather and dusty conditions may increase the risk of pterygium, only one study has been published on the pterygium prevalence in Saudi Arabia. That study was conducted in the Eastern Province, and the prevalence was found to be as low as 0.07 % [8]. However, this prevalence may be underestimated since only advanced and operable cases were included in the study. The study did not investigate pterygium management and recurrence rates of different surgical interventions.

Surgical intervention for pterygium has evolved over the past several years. Nevertheless, recurrence remains a continuous problem in every single described procedure. Previously, excision and leaving a bare sclera was the only surgical method, but this ended with recurrence in up to 88 % of the cases [9]. Multiple procedures have been described to excise pterygium, including mitomycin C injection, conjunctival autografting, amniotic membrane transplantation and beta irradiation [10]. Mitomycin C, which reduces the recurrence rate compared to a bare sclera, may cause necrotizing scleritis, scleral ulceration, endothelial cell loss and corneal edema [11–13]. The procedure of choice should provide the least recurrence with a low complication rate. Race may increase the risk of recurrence, with a study showing higher recurrence rates among Hispanic and black patients [14]. Therefore, conclusions drawn from studies conducted elsewhere cannot be applied to our population. Moreover, Riyadh is known for its dusty weather, another risk factor for pterygium formation [15]. Geographical location also plays a great role in pterygium risk, with a higher risk shown in areas of low latitude [16].

In the present study, we describe the clinical characteristics of patients presenting with pterygium. Moreover, we analyzed the recurrence rate of conjunctival autografting alone, conjunctival autografting with intraoperative mitomycin C (0.02 %), and amniotic membrane grafting over a long follow-up period. To the best of our knowledge, this is the first study in the region that compares the three different surgical procedures and analyzes their recurrence rates.

## Methods

### Sample population

 This was a retrospective cohort study that targeted patients visiting the outpatient ophthalmology clinics at King Abdulaziz Medical City (KAMC), Riyadh, Saudi Arabia. Pterygium cases coded under H110, according to the International Classification of Diseases 10th version (ICD-10), were reviewed from January 2017 to February 2020. All patients diagnosed with pterygium who had undergone an ophthalmic examination with available medical data and a sufficient follow-up period were included. A consecutive nonprobability sampling technique was used to review all cases diagnosed with pterygium over a period of three years. Initially, 320 patients were reviewed for inclusion criteria. After excluding incomplete data, a total of 292 patients were included in this study. Ninety-four patients (32.19 %) underwent surgical excision and were followed up over a mean period of 29.3 ± 11.9 months and a median of 31 months.

### Data collection

Chart medical records were reviewed for relevant historical variables, including age, gender, body mass index, and smoking status. Only cases of primary pterygium were included. The documented variables included pterygium location, corneal involvement, the presence of dry eye disease, and laterality. The modality of the surgical procedure used for a subset of patients (*n* = 94) who underwent surgical excision was evaluated in addition to the date of postoperative recurrence. Moreover, clinical comorbid conditions, including hypertension, systolic blood pressure, diastolic blood pressure, diabetes mellitus, dyslipidemia, and lipid profile, were documented. Pterygium severity and extension were defined based on the corneal involvement. The pterygium location was documented as either nasal or temporal and as to whether the cornea was involved. The presence of dry eye disease was assessed using slit-lamp biomicroscopy with corneal fluorescein staining. Body mass index was grouped into four classes: underweight (below 18), normal (between 18 and 24.9), overweight (between 25 and 30), and obese (above 30). With regard to the lipid profile, the triglyceride level was considered elevated whenever it exceeded 1.7 mmol/L, while high-density lipoprotein was considered low if it fell below 0.91 mmol/L and 1.01 mmol/L in males and females, respectively. Low-density lipoprotein was documented as high if it climbed over 4.14 mmol/L. Surgical options were divided into 3 groups: conjunctival autografting alone, conjunctival autografting combined with intraoperative mitomycin C 0.02 % and amniotic membrane grafting.

### Description of the surgical procedures

#### Conjunctival autografting

The surgery started with a subconjunctival injection of lidocaine 1 % and epinephrine with a 30-gauge needle to achieve anesthesia and hemostasis. Using forceps and Westcott scissors, the pterygium was excised from the periphery towards the limbus. Afterward, a diamond burr was applied to smooth the involved part of the cornea. Then, attention was shifted to the superior bulbar conjunctiva, and the matching size of the conjunctiva was marked and then inflated via the injection of lidocaine 1 % and epinephrine. Then, the marked area was dissected easily using forceps and Westcott scissors and was moved carefully to cover the bare sclera. The conjunctival autograft was finally sutured with 8 − 0 Vicryl sutures or occasionally with the aid of fibrin glue (Tisseel). If intraoperative mitomycin C was used, a low dose of 0.2 mg/ml (0.02 %) was applied for 1 min. Every pterygium specimen was submitted to the pathology laboratory to confirm the diagnosis.

#### Amniotic membrane grafting

The surgery started with subconjunctival injection of lidocaine 1 % and epinephrine with a 30-gauge needle to achieve anesthesia and hemostasis. Using forceps and Westcott scissors, the pterygium was excised from the periphery towards the limbus. Afterward, a diamond burr was applied to smoothen the involved part of the cornea. The amniotic membrane graft was cut into the appropriate size and then sutured to cover the conjunctival defect using Vicryl 8 − 0 sutures. Each pterygium specimen was submitted to the pathology lab to confirm the diagnosis.

#### Postoperative period

Tobramycin-dexamethasone ointment twice a day for 2 weeks along with tapering topical steroid drops for 4 weeks was prescribed. Patients were given frequent follow-up appointments in the clinic and were instructed to visit the emergency room if needed.

### Statistical analysis

Chi square and independent t tests were applied to  analyze the demographic characteristics of our patients. Categorical variables of various eye conditions are presented as proportions, and numerical variables are presented as means and standard deviations. Binary logistic regression was used to investigate factors associated with postoperative recurrence and corneal involvement, and odds ratios with 95 % confidence intervals (CIs) were calculated. Multiple logistic regression analysis was performed to adjust for age and gender. The level of significance in this study was set at 0.05.

## Results

### Baseline clinical characteristics

The study included 292 patients with primary pterygium and an average age of 53.3 ± 14.1 years. Male patients represented 76 % (222) of the sample size, and bilateral cases formed a percentage of 18.8 % (Table 1). Pterygium surgery was performed on 94 eyes. In the operated eyes, pterygium was most commonly located on the nasal side (92.6 %), and it involved the cornea in 55 % of the cases. In the univariate analysis, female gender, age > 70 years and hypertension were associated with corneal involvement (Table 2). However, in the multivariate regression analysis, female gender was the only independent risk factor (Table 3).

**Table 1:** Clinical features of patients presenting with pterygium

Characteristics			*n* = 292	
Age groups		< 50	123 (42.1 %)	
		50–70	134 (45.9 %)	
		> 70	35 (12 %)	
Mean age (years)			53.32 ± 14.12	
Median age (years)			53	
Age range (years)			18–92	
Gender		Male	222 (76 %)	
		Female	70 (24 %)	
BMI		Underweight	4 (1.4 %)	
		Normal	75 (25.7 %)	
		Overweight	102 (34.9 %)	
		Obese	111 (38 %)	
Mean BMI ±SD			28.81 ± 5.48	
Smoking Status		Nonsmoker	270 (92.5 %)	
		Smoker	22 (7.5 %)	
**Ophthalmic Characteristics**				
Eye Involvement		Unilateral		237 (81.2 %)
		Bilateral		55 (18.8 %)
Dryness				86 (29.5 %)
**Systemic Comorbidities**				
Diabetes mellitus				88 (30.1 %)
Hypertension				100 (34.2 %)
Dyslipidemia				127 (43.5 %)
**Treatment Options**				
Observation				198 (67.8 %)
Surgery	Overall operated cases			94 (32.2 %)
	CAG			19 (20.2)
	CAG + MMC 0.02 %			64 (68.1)
	AMG			11 (11.7)

*BMI* Body mass index, *CAG* Conjunctival autograft, *MMC* Mitomycin C, *AMG* Amniotic membrane graft

**Table 2:** Predictors of pterygium severity (based on corneal involvement) (*n*=94)

Factor		Cornea spared	Cornea involved	β	OR	95% CI	***P***-value
Gender	Male	37 (88.1)	33 (63.5)		1		
	Female	5 (11.9)	19 (36.5)	1.449	4.261	1.431-12.687	**0.009**
Age groups	<50	22 (52.4)	16 (30.8)		1		
	50-70	17 (40.5)	25 (48.1)	0.704	2.022	0.829-4.930	0.122
	>70	3 (7.1)	11 (21.2)	1.618	5.042	1.207-21.064	**0.027**
Smoking status	Non-smoker	36 (85.7)	59 (96.2)		1		
	Smoker	6 (14.3)	2 (3.8)	-1.427	0.240	0.046-1258	0.091
BMI	Underweight	1 (2.4)	2 (3.8)		1		
	Normal	14 (33.3)	13 (25)	-0.767	0.464	0.037-5.749	0.550
	Overweight	17 (40.5)	16 (30.8)	0.754	0.471	0.039-5.708	0.554
	Obese	10 (23.8)	21 (40.4)	0.049	1.050	0.085-12.995	0.970
Unilateral eye involvement		37 (88.1)	48 (92.3)	0.483	1.622	0.407-6.465	0.493
Dry eye disease		11 (26.2)	9 (17.3)	-.528	0.590	0.218-1.595	0.298
Diabetes mellitus		12 (28.6)	19 (36.5)	0.369	1.439	0.600-3.455	0.415
Dyslipidemia		16 (38.1)	21 (40.4)	0.096	1.101	0.478-2.533	0.821
Hypertension		10 (23.8)	24 (46.2)	1.009	2.743	1.120-6.714	**0.027**
Location	Nasal	37 (88.1)	50 (96.2)		1		
	Temporal	5 (11.9)	2 (3.8)	-1.217	0.296	0.054-1.611	0.159

*BMI* Body mass index

**Table 3:** Multivariate analysis of greater pterygium severity

Factors		β	Adjusted OR	95 % CI	*P*-value
Age	< 50		1		
	50–70	0.258	1.295	0.471–3.560	0.567
	> 70	0.856	2.354	0.485–11.435	0.617
Hypertension	Yes	0.475	2.337	0.845–6.462	0.102
Gender	Male		1		
	Female	1.273	3.572	1.125–11.339	**0.031**

### Recurrence

The overall rate of recurrence was 17 % along a mean follow-up period of 29.3 ± 11.9 months, and a median of 31 months. None of the operated patients older than 70 years developed recurrence. Young age, that is, less than 50 years, was associated with a nonsignificantly higher risk of recurrence (adjusted OR= 1.4, *p* = 0.528) (Table 4). Dry eye disease was a factor associated with an increased rate of recurrence in both univariate and multivariate analyses (Tables 4 and 5). Preoperative pterygium corneal involvement was not associated with a statistically significant increase in the recurrence rate (*p* = 0.091) in univariate analysis. Age and gender were not associated with a significant risk of recurrence in univariate analysis. Recurrence occurred in 16 eyes (17 %), with an average time of 14.2 ± 11.9 months (Table 6). The 12-month overall average recurrence rate was 10.6 %. The recurrence rates following conjunctival autografting with and without mitomycin C were 15.6 and 15.8 %, respectively. The additional use of intraoperative mitomycin C 0.02 % combined with a conjunctival autograft did not lower the recurrence rates (*p* = 0.986).  The recurrence rate following the amniotic membrane graft was twofold (OR= 2.02) (27 %) that following the conjunctival autograft (15.8 %). There was no statistically significant difference between the three surgical interventions (*p* = 0.453). In the survival analysis, there was no statistically significant difference in recurrence time between the three surgical procedures (*p* = 0.479) (Table 7). The addition of fibrin glue to Vicryl sutures did not lower the risk of recurrence (Table 8).

**Table 4:** Predictors of pterygium recurrence

Factors		Total Number	Recurrence (%)	β	OR	95 % CI	*P*-value
Age	Under 50	38	8 (21.1)	0.470	1.6	0.543–4.716	0.394
	Above 50	56	8 (14.3)		1		
Gender	Female	24	4 (16.7)	-0.034	0.967	0.280–3.342	0.957
	Male	70	12 (17.1)		1		
Smoking		8	1 (12.5)	-0.391	0.676	0.077–5.911	0.724
BMI	Underweight	3	0	-20.147	0	0	0.999
	Normal	27	3 (18.8)	-1.023	0.359	0.085–1.524	0.165
	Overweight	33	5 (31.3)	-0.667	0.513	0.148–1.785	0.294
	Obese	31	8 (50)		1		
Bilaterality		9	3 (18.8)	1.019	2.769	0.614–12.492	0.185
Dry eye disease		20	7 (35)	1.358	3.889	1.227–12.323	**0.021**
Diabetes mellitus		31	7 (22.6)	0.560	1.750	0.538–5.250	0.318
Dyslipidemia		37	9 (24.3)	0.831	2.296	0.771–6.833	0.135
Hypertension		34	7 (20.6)	0.385	1.469	0.493–4.380	0.49
Severity	Cornea involvement	52	12 (75)	1.047	2.850	0.845–9.611	0.091
Location	Temporal	7	1 (14.3)	-0.223	0.800	0.090–7.140	0.842
	Nasal	87	15 (17.2)		1		

**Table 5:** Multivariate analysis of predictors recurrence

Factor	β	Adjusted OR	95 % CI	*P*-value
Age Above 50	0.360	1.433	0.468–1.433	0.528
Dry eye disease	1.321	3.748	1.174–11.961	**0.026**

**Table 6:** Details of overall recurrence rate

Recurrence Rate	
Overall postoperative recurrence	16 (17 %)
Recurrence before the first year	10 (62.5)
Recurrence after the first year	6 (37.5)
Mean time of recurrence in months	14.19 ± 11.90
Median of time of recurrence in months	12
Range of time of recurrence in months	2–40

**Table 7:** Recurrence after each surgical procedure

Surgical procedure	Total number of cases	Recurrence (%)	β	OR	95% CI	***p***-value	Survival Analysis		
							Mean time of recurrence in months ± SD	95% CI	***p***-value
Conjunctival autograft	19	3 (15.8)		1			8.67± 3.33	2.13-15.2	0.479
Conjunctival autograft + MMC	64	10 (15.6)	0.012	0.988	.242- 4.028	0.986	13.5 ± 3.83	5.99-21.01	
Aminiotic membrane transplant	11	3 (27.3)	0.693	2.02	0.472- 8.634	0.453	22 ± 8.72	4.91-39.09	

**Table 8:** Recurrence and survival rates following each surgical procedure depending on closure technique

Procedure (=92)	Conjunctival Autograft (***n***=18)					Conjunctival with MMC (***n***=63)					Amniotic membrane transplant (***n***=11)				
Closure technique	Recurrence		Survival analysis			Recurrence		Survival analysis			Recurrence		Survival analysis		
	n (%)	P	Mean±SD (months)	95% CI	P	n (%)	*P*	Mean±SD (months)	95% CI	*P*	n (%)	P	Mean±SD (months)	95% CI	*P*
Sutures alone (n= 62)	2 (20)	0.671	7 ±5	0 – 16.8	0.405	6 (14)	0.541	16.33±5.92	4.73-27.94	0.872	3 (33.3)	0.338	22±8.72	4.91-39.09	NA
Suture + fibrin (n= 30)	1 (12.5)		12	12		4 (20)		9.25±3.54	2.30-16.19		0 (0)		NA		

2 patients underwent closure by fibrin alone and had no recurrence

## Discussion

This is the first study Saudi Arabia to describe the characteristics and recurrence of different surgical methods over a long follow-up period. We found that pterygium was most commonly located on the nasal side. Different theories surround the tendency of pterygium to occur nasally. First, the protective effect of the nose may protect the temporal side against UV light shining nasally [17, 18]. Second, dust particles flowing nasally into the nasolacrimal duct may cause mechanical irritation [17, 19]. Finally, it has been proposed that forehead perspiration with lactic acid contained in sweat falling from the forehead downside the nose into the nasal conjunctiva may trigger biochemical irritation [17]. Pterygium was found to be most common in people between 50 and 70 years old. Multiple studies have found that age greater than 50 years is a risk factor [7, 20, 21]. It is expected that increasing age increases people’s exposure to UV radiation. In our study, pterygium was found to be more common among males. This might be due to differences between the two genders in Saudi Arabia in terms of time spent outdoors. Studies in South Korea found that male gender was a risk factor [21]. In Indonesia, there was no difference between males and females in developing pterygium [22]. Notably, only 7.5 % of our patients were current smokers. Meta-analytical studies have identified smoking as a protective factor against pterygium [7, 23]. The underlying molecular protective mechanisms of smoking are unknown. In the present study, the rate of bilateral pterygium was 18.8 %. This is close to the rate reported in Inner Mongolia, which was 24.2 % [20]. The only published study about pterygium in Saudi Arabia reported a higher rate of bilateral pterygium (46 %), probably because only advanced pterygia were included in that study [8].

Corneal involvement was observed in 55 % of the operated eyes. Previous studies have shown that pterygium causes not only astigmatism but also high-order aberrations with larger pterygia [24, 25]. In our study, the only independent risk factor for corneal involvement was female gender. Tan et al. developed a grading system based on the clarity of blood vessels and eventually found that it is a risk factor for recurrence following bare scleral excision [26]. Most previous studies graded pterygia based on their morphological appearance and not on their anatomical extension [22, 26–28]. Studies on pterygium severity, based on pterygium blood-vessel clarity, found that the only risk factor for greater severity was sun exposure for more than 5 h per day [27]. In another study, male gender and outdoor activities were found to be risk factors for fleshy pterygium [28]. In our study, we graded pterygium anatomically, and corneal involvement was not found to be associated with a significant risk of pterygium recurrence. A previous study showed that corneal involvement increased pterygium recurrence; however, the sample was limited to only male patients undergoing bare sclera excision with intraoperative mitomycin C 0.02 % [29].

 In the present study, none of the patients older than 70 years (14 patients) developed any recurrence. In multiple studies, young age was shown to be a risk factor for recurrence after a limbal conjunctival autograft [30, 31]. In a similar manner to what we observed, patients under the age of 54 years were found to have a higher rate of recurrence following conjunctival autografting, albeit this difference was not statistically significant [32]. This finding can be explained by the fact that aging slows and delays the wound healing process [33]. In other studies, age was not found to be a risk factor for recurrence [34]. Noticeably, dry eye disease was found to be the only significant risk factor for an increased recurrence rate. Dryness triggers inflammation by increasing matrix metalloproteinase (MMP)-9 in the tear film [35, 36]. In fact, it has been suggested that MMP-9 is a useful biomarker in the diagnosis and follow-up of patients with dry eye disease [35]. Yang et al. found that MMP-2 and MMP-9 are expressed in advanced pterygia [37]. Interleukin-6 (IL-6), which is elevated in patients with dry eye disease, was also shown to be significantly higher in cases of recurrent pterygium compared to primary pterygium [38, 39]. Frequent postoperative lubrication is helpful for decreasing these proinflammatory cytokines. Additionally, artificial tears provided additional benefit to topical steroids in reducing the rate of pterygium recurrence [40]. On the other hand, pterygium recurrence was also found to increase dryness and decrease the tear osmolarity 12 months after recurrence [41].

Recurrence remains a challenging problem in pterygium management. The overall recurrence rate in our study was found to be 17 %, with an average time of 14 ± 11.9 months, with 37 % of cases occurring after the first year. This stresses the importance of long-term follow-up after pterygium excision. With regards to conjunctival autografting, a procedure first described Kenyon et al. in 1998 [2], recurrence was noted in the present study in 15.8 % of the cases. This is close to what has been shown by Huerva et al., who reported a recurrence rate of 11.76 % over an average follow-up time of 49 months [42]. In a study with a similar mean follow-up period (34 months) to that in our study (29 months), recurrence occurred in 11.4 % of cases [43]. In another study on conjunctival autografts, recurrence was observed in 4 cases (rate of 8 %), with an average time of recurrence of 4.5 months [32]. We assume that we demonstrated a higher recurrence rate in our study due to geographical differences. Our study was conducted in Riyadh, a city for its dry and dusty weather conditions, which is a risk factor for pterygium development and may be responsible for raising the risk of recurrence. Moreover, our rate was higher possibly because we had an extended follow-up period. Another study showed a low rate of recurrence (10 %) because of the short-term follow-up (14 months) [44]. As a general overview, the recurrence rate following conjunctival autografting ranged from 5 to 39 % [9, 42, 44–48]. The variation in recurrence rates is mainly due to differences in follow-up periods, study populations and areas. Not only is this point salient, but surgical skills and experience may also play a role in the fluctuation of recurrence rates. Previous experience with conjunctival autograft procedures was significantly associated with lower recurrence [46]. The mean overall recurrence rate over 12 months in a recent study on primary closure, conjunctival autografts, and amniotic membrane transplantation was 11.3 % [49]. This is similar to the 12-month overall rate of recurrence in the present study, which was 10.6 %. Similar rates of recurrence were observed in the present study between conjunctival autografts with and without mitomycin C. Similarly, in a retrospective observational study, mitomycin C 0.04 % for 2 min did not lower the risk of recurrence [31]. In a study on conjunctival autografts combined with mitomycin C, the recurrence rate was 3.6 % [50]. However, the study was only conducted on 28 patients over 12 months, which may have underestimated the recurrence rate. In a larger retrospective Japanese study, the recurrence rate after conjunctival autografting with intraoperative mitomycin C was 3.96 %; however, the follow-up time was only 9 months [48]. In the present study, recurrence following conjunctival grafting with intraoperative mitomycin C developed in an average time of 13.5 months. Fibrin glue was occasionally added to Vicryl sutures to improve patients’ postoperative comfort and reduce graft-related complications [51]. In two meta-analytical studies, a lower recurrence was noted with fibrin glue than with sutures [52, 53]. When fibrin glue was added to sutures in our study, the recurrence rate was similar with no statistically significant difference. In a recent study on pterygium recurrence, patients were divided into 3 groups: fibrin glue combined with conjunctival sutures, fibrin glue alone and sutures alone. The study did not show a statistically significant difference between the three groups [51]. However, the number of recurrences was too small to find any differences. More studies are needed to confirm whether fibrin glue applied over Vicryl sutures can reduce pterygium recurrence.

The amniotic membrane promotes conjunctival epithelialization and inhibits fibroblasts [54]. The advantages of amniotic membranes include short postoperative visual recovery, better comfort and less operation time [55]. Moreover, the amniotic membrane may cover a large area of the defect or be utilized when conjunctival scarring exists, which prevents sufficient autografting. In the present study, recurrence was higher in the amniotic membrane (OR= 2.02) (27 %) than in the conjunctival graft (15.8 %) of the cases treated with an amniotic membrane graft. The range of recurrence rates following amniotic membrane grafts reported in the literature ranges from 14.5 to 41.7 % [55–60]. In a study comparing conjunctival autografts with amniotic membrane grafts, the rates of recurrence were 13.1 and 28.1 %, respectively, over 6 months [61]. Our study identified similar recurrence rates, but the difference was not statistically significant because of the relatively low number of amniotic membrane grafts.

Because of the retrospective nature of the study, it was difficult to report the exact sizing and morphological description of the lesions. Also, data on pterygium severity was available only for patients who underwent surgical excision and missing for patients who did not undergo excision. Since all our patients share the same race, it was not possible to analyze whether race is a risk factor for recurrence. Long-term multicenter prospective studies on pterygium recurrence are required to analyze risk factors for recurrence. The present study did not include a healthy control group, which prevented us from calculating the risk factors and prevalence rate of pterygium; however, this was not part of the objectives of the study. Future studies on pterygium recurrence may be conducted by one surgeon to control for surgical experience factors.

## Conclusions

In conclusion, the only factor associated with the recurrence of pterygium was dry eye disease. More than one-third of recurrences developed after the first year, which stresses the importance of long follow-up. The recurrence in our study following conjunctival graft was slightly higher compared to the literature mainly due to differences in study area, population and follow-up periods. Our study identified similar recurrence rates following conjunctival autografting with versus without mitomycin C. Nevertheless, the recurrence rate following amniotic membrane grafting was twofold that following conjunctival autografting. However, the difference was not statistically significant because of the relatively lower number of patients who underwent amniotic membrane transplantation.

## Data Availability

The datasets used and/or analysed during the current study are available from the corresponding author on reasonable request.

## Abbreviations

UV: Ultraviolet
CI: Confidence interval
BMI: Body mass index
MMP: Matrix metalloproteinase
